# Patients With Cardiovascular Disease Revisiting Specialist Physicians via Remote Treatment: Interview Study of Experiences

**DOI:** 10.2196/43125

**Published:** 2023-06-01

**Authors:** Charlott Ek, Per-Daniel Liljegren, Anette Edin-Liljegren

**Affiliations:** 1 Jokkmokks Healthcare Centre Region Norrbotten Jokkmokk Sweden; 2 Primary Care Region Västerbotten Umeå Sweden; 3 Department of Epidemiology and Global Health Umeå University Umeå Sweden

**Keywords:** remote treatment, sparsely populated region, telemedicine, content analysis, experiences, person-centered care, rural, eHealth, mobile phone

## Abstract

**Background:**

Access to health care for an aging population with growing needs presents major challenges in northern Sweden’s sparsely populated regions. Few people, the lack of professionals, and long distances make it difficult to provide health care on equitable terms according to the Swedish legislation. Remote treatment (RT) using information and communication technology has been suggested to overcome these difficulties, and person-centered care (PCC) is a desired philosophy to improve the quality of health care. However, there is scarce knowledge about how patients experience RT meetings.

**Objective:**

This study aimed to describe the experiences of patients with cardiovascular disease revisiting specialist physicians via RT guided by a PCC perspective in northern Sweden’s sparsely populated regions.

**Methods:**

A qualitative study was conducted based on interviews with 8 patients with cardiovascular disease revisiting their physician through RT, from a digital health room to a health care center or from a health care center to a hospital. The interviews were recorded, transcribed verbatim, and analyzed using inductive content analysis. The results are discussed from a PCC perspective.

**Results:**

The analysis resulted in 6 categories: good accessibility, safety with good relationships, proximity and distance with technology, habit and quality of the technology facilitating the meeting, cherishing personal integrity, and participation in own care. These categories were interpreted as the theme, *participation and relationships are important for good and close care via RT*.

**Conclusions:**

The study shows that *participation and relationships are important for good and close care via RT*. To improve the quality of an RT meeting, PCC can be applied but needs to be extended to the digital domain—electronic PCC, especially the communication component, as it is the most salient difference from a face-to-face meeting. Important factors that should be considered before, during, and after the RT meeting have been identified.

## Introduction

### The Challenges of Health Care in Sparsely Populated Regions

Sparsely populated regions (SPRs) have many things in common across the world such as low population density, long distance to health care and other societal services, being governmentally remotely controlled, and a distinct lifestyle [1]. These regions are not only different from urban areas but also different from rural regions in general and have been described as a specific geographic category comprising >60% of the Earth [1]. Therefore, it is important to study access to health care under these conditions. In the SPR of northern Sweden, demographic transition and urbanization have led to a large proportion of older adults still living in their homes [2]. The geographical location with long distances to health care units makes access to care challenging [3-5], and studies show that people living in SPRs receive poorer care than those living in cities [5], which is contradictory to Swedish law where “the goal of healthcare is good health and care on equitable terms for the entire population” and the care should be organized close to the people [6]. There have been several highly prioritized initiatives from the Swedish government [7] to ensure good-quality, local health care; however, it is still unclear how this should be implemented in SPRs.

### Opportunities With Digital Technologies

A way to overcome these challenges is to use information and communication technology (ICT), which has been recommended and encouraged by the World Health Organization [8]. An initial statement from the World Health Organization Bellagio eHealth Evaluation Group proposed that “To improve health and reduce health inequity, rigorous evaluation of eHealth is necessary to generate evidence and promote the appropriate integration and use of technologies” [8].

It is important to evaluate the implemented methods and techniques because despite its many benefits, the introduction of new technology may lead to new problems, such as patient integrity and safety issues [9]. In a Danish Island, more than half of the patients did not like consulting a specialist via ICT [10]. In the study, a large proportion of older adults and people with only primary education indicated that there could be difficulties in introducing ICT in rural areas where the level of education is lower, in general, than in urban areas. In a study by Call et al [11], overall, 43% of the participants were still averse to telemedicine despite the inconvenience of in-person visits. Recently, the COVID-19 pandemic has further enforced the use of telemedicine [12]. However, there is both a lack of consensus of terminology and a knowledge gap regarding how various aspects of telemedicine work.

The level of education, previous use of social media and other communication platforms, and being a rural resident are factors that may influence how receptive the informants are to telemedicine [10,11]. Age is correlated negatively to computer literacy [12,13], which affects the outcome of the introduction of technology. In contrast, several studies have shed light on the importance of telemedicine from the perspective of patient satisfaction [5,14-16]. Patients reported saving time and reducing costs by not having to travel and were satisfied with the technical performance [17-20]. Furthermore, some patients who were negative about using video meetings initially changed their minds when they tried it [15]. Therefore, there is reason to believe that follow-ups of planned care visits via ICT could be a valuable complement to physical meetings when physicians and patients have already established a relationship.

To provide more qualified care for people living in SPRs, remote treatment (RT), which we define as *treatment that is conducted remotely by means of ICT, including medical advice, examination or treatment, where the patient and therapist are separated in space, but not in time*, may be an option. Thus, we considered RT as a subset of the broad concept of telemedicine to limit and clarify the aim of this study [21].

RT is also important for sustainable health care and is likely to be of great benefit for patients, professionals, caregivers, and society and is a way of increasing accessibility to health care on equitable terms and supplying specialized skills to remote areas. Therefore, there is a great need for systematic studies to ensure the quality of RT meetings and to obtain patient experiences of safety, partnership, and shared decision-making. Thus, RT can save both time and money, primarily for patients who must travel long distances to health care units, and can also reduce the carbon dioxide footprint of health care [3,22].

As cardiovascular diseases are among the most common diseases in the world and in Sweden [23], it is important to increase knowledge about these patients’ experiences of seeing their physician for planned follow-up meetings via RT and how telemedicine solutions can be a way to increase access to health care in SPRs.

### Person-Centered Care—A Desired Model

Person-centered care (PCC) is a care philosophy that aims to include the life-world perspective and seeing the whole person and has been developed to improve the quality of health care [24,25]. A transition from a care model with the patient being seen as passive to being active in their own care and their own resources used are important factors [24]. PCC creates a sense of self-empowerment to manage one’s own illness; contributes to safety; and is linked to short care times, few readmissions, and better quality of life for the patients [26]. PCC is a collaboration and a partnership between the health care staff and the patient. It is a mutual approach in which health care professionals respect the knowledge that the patient can provide about their own life and health situation, such as values, goals, and previous experiences. The health care staff contribute with their professional expertise and information about care alternatives [26].

PCC means that “individuals’ values and preferences are elicited and expressed, guide all aspects of their health care, supporting their realistic health and life goals” and is achieved through a dynamic relationship between individuals and health care professionals [27]. Recently, PCC has been proposed as a desired care model in several Swedish governmental reports, which is a step toward legislation [7,23,28].

The rapid development of digital technologies and the need for transformation of the health care system make PCC a natural starting point for investigating RT.

Thus, both RT and PCC have been suggested to improve the accessibility, efficiency, and effectiveness of health care [7,24,29]. However, there is still a lack of knowledge about how patients experience RT meetings.

This study aimed to describe the experiences of patients with cardiovascular diseases regarding follow-up meetings with their physician through RT, in northern Sweden’s SPR, guided by a PCC perspective.

## Methods

### Study Design

A qualitative approach was used to reflect the experiences of people who receive RT. The data were originally collected in a master thesis at the Department of Nursing at Umeå University and were further analyzed in this study. According to the guidelines for necessitating quality and transparency of health research, Consolidating Criteria for Reporting Qualitative Research (COREQ) [30] were followed during the process.

### Participants and Settings

**Figure 1:**
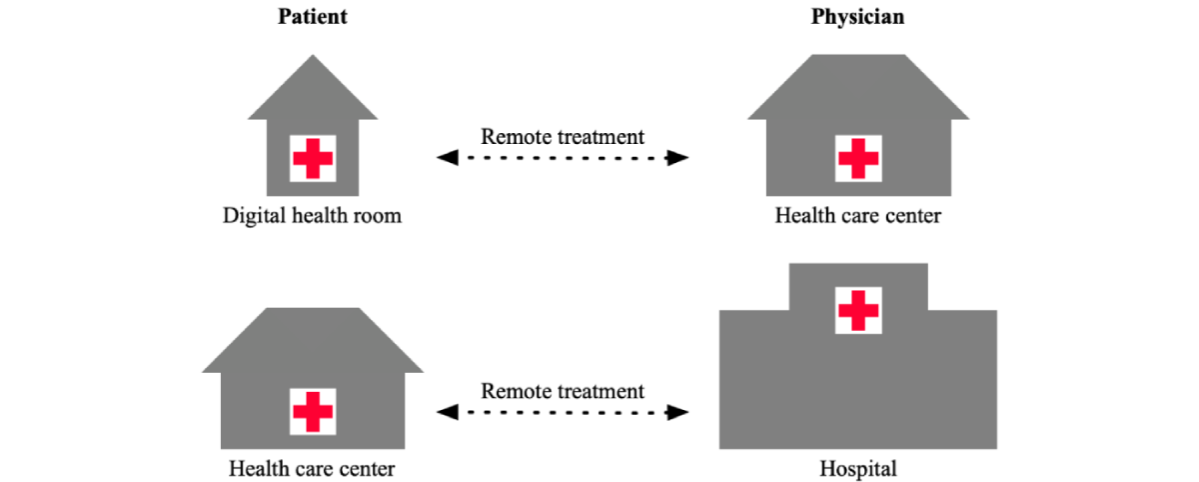
Settings for remote treatment.

This study was conducted in the SPR of northern Sweden, where Region Västerbotten and Region Norrbotten are official health care providers. RT was conducted with a patient and a specialist physician having a digital meeting between a health care center (HCC) and a hospital—or between an HCC and a digital health room (DHR; Figure 1).

The DHR is a room equipped with ICT, an encrypted videoconferencing system, and other medical devices that are not available at home. The DHR has been established in small villages in Västerbotten County, close to the inhabitants, to provide more accessible and equitable health care. In this remote area, the distance to the nearest hospital could be >300 km. Before and during some of the meetings, the staff was sampling, performing examinations, and supporting the patients with connection to the videoconference system. A digital stethoscope was used to transmit heart and lung sounds in real time to the connected medical specialist during the visit for some patients. The operation manager from the HCC and a nurse at the hospital recruited participants for this study. The inclusion criteria were patients aged >18 years diagnosed with cardiovascular disease who have had a planned revisit to their physician via RT. Participants were informed in writing and orally about the study and asked whether they would participate, and they signed an informed consent form before the interview started. The 8 participants consisted of 4 (50%) women and 4 (50%) men, aged 53 to 85 years (Table 1).

**Table 1:** Patient characteristics and way of connection to specialist physician from a digital health room (DHR) to a health care center (HCC) and from an HCC to a hospital.

Patient ID	Age (years)	Sex	RT^a^ connection
1	83	Female	DHR to HCC
2	85	Male	DHR to HCC
3	81	Male	DHR to HCC
4	72	Female	HCC to hospital
5	75	Male	HCC to hospital
6	68	Female	HCC to hospital
7	78	Male	HCC to hospital
8	53	Female	HCC to hospital

^a^RT: remote treatment.

### Data Collection

The data collection was inspired by the PCC philosophy, but because there is no unified theory [25], we constructed open-ended questions [31] about people’s experiences of meeting their physician at a distance. Data were collected using semistructured interviews by the author (CE). The questions were about what worked well and what did not in the digital meetings, relations, experiences of connection to the physician, differences between physical and digital meetings, what it means to get access to digital meetings, and how the meeting could be improved. Each question was followed by further questions to develop previous statements and encourage the interviewee to talk more about the situation and give examples. The interviews were conducted at the participants’ homes, recorded digitally, and transcribed verbatim. As it was difficult to find participants, the study and the interviews were conducted over 2 periods—during July 2017 and from November 2018 to February 2019; the interviews lasted 22 to 42 (median 29) minutes.

### Data Analysis

The transcribed text was analyzed systematically using content analysis described by Graneheim and Lundman [32]. The results were then interpreted inductively, which is recommended in the literature if the knowledge gap of what will be studied is limited or fragmented [33]. Furthermore, the interviews were read through carefully several times to get a sense of the whole of the material. Text units with the corresponding purpose of the study were chosen and condensed. The text units were coded close to the text, which were then abstracted into subcategories. Furthermore, subcategories that were similar to each other were sorted and abstracted into categories. The categories related to each other, and the underlying sentences were interpreted and formulated in a theme such as descriptions as a common thread, where the sentence reappeared in category after category [33]. Overall, 2 authors (CE and AE-L) discussed codes, subcategories, categories, and the theme with each other during the analysis process to ensure the credibility of the study [34,35].

### Ethics Approval, Informed Consent, and Participation

The study was conducted in accordance with the ethical principles described in the Declaration of Helsinki [36]. In SPRs, one needs to be careful about ethical issues when presenting data about patients. In a small village, even age and sex in combination with a medical condition may be sensitive data for identifying a person. Informed consent was obtained from both the operation managers and the participants. Participants were informed both in writing and orally about the possibility to participate in the study and that they could cancel their participation at any time without providing any reason [36]. Participants were also informed that personal information and data from the interviews could not be attributed to the individuals and that they have been treated confidentially. The study was approved by the Regional ethical review board located in Umeå (2017/155-31 and 2018/237-32).

## Results

### Overview

The analysis of the interviews resulted in 6 categories: good accessibility, safety with good relationships, proximity and distance with technology, quality of and familiarity with technology facilitating the meeting, and cherishing personal integrity and participation in care. The categories were abstracted and sorted from a total of 16 subcategories, as shown in Textbox 1. From the categories, a theme was interpreted as *participation and relationships are important for good and close care via RT*. The categories and the theme are presented in the following sections and illustrated with quotes from the interviews.

**Overview of categories and subcategories of the theme participation and relationships are important for good and close care via remote treatment.**

**Good accessibility**

- Traveling and safety
- Time saving
- Equitable care

**Safety with good relationships**

- Familiarity with the staff
- Comfortable togetherness in the waiting room
- Relatives provide support

**Proximity and distance with technology**

- Being close and feeling distance via the video screen
- Personal and impersonal contact
- Calm and focused meeting

**Quality of and familiarity with technology facilitating the meeting**

- Being familiar with the technology makes the meeting easy
- Supported or disturbed by technology

**Cherishing personal integrity**

- No public self-disclosure
- Importance of privacy

**Participation in care**

- Being prepared
- Wanting more information
- Opportunities for development

### Good Accessibility

The informants expressed that it was valuable to reduce the time spent in traveling to revisit their physician and to increase safety by not having to drive. The possibility to meet the specialist via RT was perceived as more equitable care and was interpreted as a common category, *good accessibility*.

#### Traveling and Safety

Participants in the study experienced a great advantage in avoiding traveling, thus reducing the amount of driving. It was convenient and easy with the short route, or the health care unit was so close that the informants could walk to the meeting instead of traveling long distances and seeing the physician for just a short time. Although the interviewees living in SPRs were used to traveling long distances, in winter, with difficult and unpaved roads, it was especially valuable to avoid traveling. The individuals also experienced that the evening sun in the eyes could be tiring when driving. Owing to the northern location being close to the Arctic circle, during the winter season, the sun is very low to the east in the mornings and very low to the west in the afternoons and thus in the eyes—both ways to the HCC unit and home:

...I thought it was great because then you do not have to go to [the hospital] and get away from driving and all that...and because it is so close it is only a couple of minutes to walk there...

ID6; female; aged 68 years

Some informants found it difficult to drive far owing to illness and pain. Reducing travel also meant an economic advantage, as they used their own car to drive to the HCC or hospital:

...Say that it cost 150–200 SEK in fuel for a trip and then you get 24 SEK for it in compensation...it is not worth it...

ID8; female; aged 53 years

Even when it was easy to go to the health care unit, the taxi ride to the hospital or HCC could be agreeable with drivers you know. Despite long distances, the journey could be pleasant, and sometimes, it was not difficult to travel, especially when informants also took the opportunity to go shopping or do other errands at the same time:

Even so, a trip to [the city] means you can go to the shops before and after and do errands there.

ID6; female; aged 68 years

#### Time Saving

Some participants indicated that having the meeting with the specialist via RT saved time and that they received help more quickly and avoided worries. RT made the whole day easy and took just 15 minutes compared with the fact that it takes half a day to see a physician in a hospital. Participants felt that time was saved for both themselves and the physicians. Time was also saved for the staff, whose job is to support with connections and keep track of the routines so that the physician’s visit was not delayed. An interviewee said that everyone has the same amount of time and we live in a stressed society; therefore, it was good that care could be provided remotely for people with long distances to health care units. A participant of working age saw benefits in not having to take time off, not missing working hours, and earning income:

And if you work and...I do not have to miss so much working time and do not keep on and may not need to compensate for work so much and make changes with colleagues...so there is not much lost work income either...there are financial benefits...

ID8; female; aged 53 years

#### Equitable Care

Participants in the study felt that the care was equitable and that they received the same assessment as at the HCC or at the hospital for this type of revisit. The RT meeting felt normal and was not different, except that the physician and the patient were not physically in the same room. If they had gone to the HCC or to the hospital to meet the physician face-to-face, the physician would have asked the same questions as asked during the digital meeting:

The great thing is that it is equitable...what should I say...it gives just as good results with these technical facilities [video].

ID2; male; aged 85 years

### Safety With Good Relationships

In the interviews, it was noted that the patients were familiar with the staff and experienced a comfortable time together in the waiting room, including relatives who gave support in the meeting with the physician, which was interpreted as *safety with good relationships*.

#### Familiarity With the Staff

Participants in the study were familiar with the staff at the health care unit and already knew the physician before the meeting, which created security and a feeling of safety in the meeting. The informants thought that the physician was pleasant and easy to talk to during the meeting. A participant knew his physician only through phone before but felt that the meeting worked very well. It was very important that they had met the physician before, instead of meeting a new unknown physician who did not know anything about them as a person:

But had it been a complete stranger then you get a little...then you keep a little distance...if you...you think what the heck is this.

ID7; male; aged 78 years

#### Comfortable Togetherness in the Waiting Room

A good social gathering was experienced when the patients met acquaintances in the waiting room at the HCC unit. It became a pleasant meeting place, similar to going to the neighbor’s house and meeting people you know. The conversations were relaxing, and there were discussions about what had happened since the last time they had met, how it was on the fishing trip, and even some talk about illness. A participant thought that it felt similar to home and he could be himself:

...We were standing out there talking and then we entered and then you could have coffee if you wanted and another acquaintance was sitting there and it was no big deal to get there as dressed as when you walk in a village, it feels like home in some particular way, yes...

ID1; female; aged 83 years

#### Relatives Provide Support

Close relatives could be a great support in the RT meeting, which provided a feeling of safety both before and during the meeting. Participants experienced that close relatives were a support when the patient had hearing or memory problems, for example, after a stroke:

And so I had [the man] was there to support me if I forgot something or if I lost words...

ID6; female; 68 years

### Proximity and Distance With Technology

Both proximity and distance were experienced in the RT meeting, a contact that could be perceived both as personal and impersonal. The meeting felt calm and focused on the patient themself, which was interpreted as *proximity and distance with help of technology*.

#### Being Close and Feeling Distance via the Video Screen

The image on the screen was large and clear, and the patients were affirmed by the physician on the screen. Participants felt that it was as close as in real life, almost similar to sitting in the same room. Some informants perceived it as if the physician was behind the video screen and that they had eye contact. The patients experienced that the physician could see their reactions and facial expressions:

That he or she can look at my face and could see how I think before I answer, I actually think if I’m honest or making up [laughter]...that’s exactly what I think I can do with the grandchildren when I talk to them on Skype.

ID1; female; aged 83 years

The interviewees experienced that the sound was good when speaking to the physician, without interruption, and that it was easy to communicate. A person described how they went through the medication list in the RT meeting. The patients were also impressed by the possibility that the physician could zoom in on them and listen to their heart and lungs through the digital stethoscope:

They listen to my heart and then hear all that, 75 km away, so the physician can sit and listen to my heart, it’s so amazing it’s crazy. Ahh...I think that’s really impressive.

ID1; female; aged 83 years

Also, a feeling of *distance* was experienced by some informants in the RT meeting. It felt different, and some participants found it difficult to be spontaneous and answer the physicians’ questions. For them, it was difficult to see body language and facial expressions:

If I talk to a person sitting in front of me, I can see their body language, I can joke with the person...I can ask and say things that are almost private but a person who is on a screen is a bit distant because I feel like I can’t really talk.

ID 6; woman; aged 68 years

The feeling of absence of physical contact was experienced when the physician could not touch the patient and measure the pulse. They thought that physical contact should be the right way to meet the physician, because people become more sensitive if they are close to each other. Some patients were still satisfied and thought that the meeting was normal without physical contact and that the on-site nurses could do the examination. However, a participant wanted a physical meeting:

They never asked me what I wanted they just said it would be through video but I would have preferred to meet them there [at the hospital].

ID4; female; aged 72 years

#### Personal and Impersonal Contact

Patients in the study found the personal contact to be perfect even though they met the physician via a computer screen and they were not physically in the room:

It was like personal contact even though it was via such a link.

ID7; male; aged 78 years

Some patients felt the opposite, that the physician’s visit was impersonal via RT, and they experienced a feeling of insecurity. Some other participants had problems in getting something out of the meeting; they did not ask their questions because the meeting did not feel personal. A person wanted to meet the physician physically because he had vision and hearing problems, which resulted in the physician feeling like a stranger in the RT meeting. It became uncomfortable; therefore, the person barely remembered the meeting and thought it was something wrong with her but said that the physician was certainly professional:

I would rather have a personal meeting, you can reach them in other ways when you have vision problems and sitting close because I would like to comment on things under...and when they were like strangers to me, I couldn’t.

ID4; female; aged 72 years

Another participant expressed that although the meeting did not feel personal, it worked to meet the physician via the video screen:

It’s not this kind of personal, so I feel, but I thought it worked well.

ID7; male; aged 78 years

#### Calm and Focused Meeting

The RT meeting was experienced as calm and focused and almost as in a home environment. The patient got easy contact with the physician in a peaceful and quiet way, and nothing was disturbing from the background. The informants thought that the physician was responsive and gave them time to ask questions. This meant that the patients did not feel stressed and felt that the physician was focused on them during the meeting. At an ordinary physical meeting with the physician, people look around at things on bookshelves and other things in the room, but all that disappeared in the RT meeting, which was perceived as positive. A person got the feeling that the meeting was focused as only one could talk at a time:

I don’t know if it was because of the technology...You have to be quiet, it felt that way anyway...When one talks, you listen to what he will say...But I don’t think it’s something negative...then you get even more focused than maybe talking at the same time...because then neither of us really listens.

ID8; female; aged 53 years

### Quality of and Familiarity With Technology Facilitating the Meeting

The informants experienced that technical skills facilitating the RT meetings but that technical quality could both support or disturb the meeting, resulting in the interpretation of the category, *quality of and familiarity with technology facilitating the meeting*.

#### Familiarity With the Technology Makes the Meeting Easy

The interviewees felt that the physicians gave the impression that they were comfortable with the technology. In addition, the patients themselves felt comfortable. It was not strange because they were used to the technology related to using the internet, Skype, or other systems through their work:

But you’re used to watching TV so you’re not completely alienated from things like being on Skype with grandchildren on the iPad.

ID1; female; aged 83 years

Some participants also felt unfamiliar and insecure when they had a digital meeting. They knew it was possible to meet the physician via RT but had not had any meetings themselves before. The first time felt special, strange, and stiff because everything was new but, at the same time, exciting. The informants experienced the feeling of not having control, but after a while, they got used to it:

And then the physician came, and it felt a little bit strange when you’re not like this [Physically]...but you see a TV screen, but after we had been sitting for a while there was nothing strange about that.

ID7; male; aged 78 years

There were participants who felt old-fashioned when they would talk via a video screen, but they thought it was a matter of age and habit. Participants hoped for more opportunities to have RT meetings, and they imagined that when they got used to it, it would feel similar to sitting in front of a physically present physician. In addition, a person felt that the physician was uncomfortable with the technology:

I think she was uncomfortable in front of...I wonder if she’s done this before. I’m not sure about that...because I found it uncomfortable for her to sit in front of the screen.

ID6; female; aged 68 years

#### Supported or Disturbed By Technology

Some interviewees told us in the interview that they got support and help from the staff to start the meeting. The informants also mentioned that when they arrived at the HCC unit, they were directed to a room with a table and a video screen, and the staff started the computer and instructed them about how to use it. In the RT meeting some participants also mentioned how the physician instructed the staff about how to put the stethoscope in place to be able to listen to the sounds from the heart and lungs. An informant said that the physician informed them how the videoconference would be conducted:

...She told me where it was and that she was going to ask me a few questions...And I said it’s just to ask questions...I’ll answer as best I can. What I understood, it went as well as possible.

ID5; male; aged 75 years

Overall, the technology worked well, but participants felt that the technique could be disruptive. A participant said that it was a hassle with the sound and it was difficult to hear the lung and heart sounds using the digital stethoscope, but it started to work at the end of the meeting. Another participant could only see half of the physician’s face and thought it felt strange:

...At last she got herself on the screen but it just happened that we only saw half of her, I think we saw her from the nose and upward so she sat like in a corner of the picture, and then we talked to her but it felt quite strange to sit and talk to half a person, half a face.

ID6; female; aged 68 years

### Cherishing Personal Integrity

The patients felt that they did not want to disclose information about themselves at the digital meetings and that it was important to have a private room when they met the physician, which was interpreted as *cherishing personal integrity*.

#### No Public Self-disclosure

Participants in the study said that several individuals were present during the RT meeting and that they did not want to disclose themselves to people other than the physician. Other people could be present because the health room was also used as a gathering point for the home care service in the area. Participants thought that it was a sensitive situation, and they did not want other people to hear what thoughts, worries, and illnesses they had, even though they knew the staff had a duty of maintaining confidentiality:

I may not want so many people listening and hearing what I’ve been thinking about, what illness, what thoughts and what problem I have or concern...

ID1; female; aged 83 years

#### Importance of Privacy

Participants pointed out the importance of individual meeting rooms and that people should not pass by all the time. When people passed by, it was difficult to focus on the meeting and maintain confidentiality:

Then you sat alone in your own secluded room and it is also quite important.

ID8; female; aged 53 years

### Participation in Care

The informants had a desire to be prepared for the meeting with the physician, wanted more information and influence, and saw opportunities for the development of care. These are summarized in the category *participation in care*.

#### Being Prepared

Participants had written down questions and thoughts they had—such as medications, how they would think ahead, and future follow-ups—and they wanted to be prepared for the RT meeting. It was important to be prepared; otherwise, they had the risk of forgetting half of their thoughts:

I probably got answers to all the questions I had; I had written down what to ask for and she answered...

ID6; female; aged 68 years

#### Wanting More Information

There was a request from the participants for more information before the RT meeting. As first-time users, they had heard of an appliance they could talk to and thought that someone would be there to give support and tell them how it worked practically. A participant was concerned that the screen was not switched off after the visit:

I felt awfully bad because I thought now it’s on...what if...A lot of these, what if...and standing in [the hospital]...and what if the power is on? All that practical stuff...I walked around and thought about it for a long time.

ID6; female; aged 68 years

There was a desire for information before the appointed meeting, so that everything could be arranged properly. The participants wondered whether there were any routines at the HCC unit when they got the feeling that no one knew anything about the meeting, for example, who shows the patient the way and who would initiate the meeting via the video screen. A person lacked information and felt that she was not involved in the visit, and she wanted someone to coordinate the visit:

But now I understand that it’s an expense for the healthcare system but in this particular case it would only have been the cost of one trip that I would have anyway when I was going to [the hospital].

ID4; female; aged 72 years

#### Opportunities for Development

Joy and hope for the future were expressed by the participants. Some informants experienced the meeting as fantastic and wished to continue conducting RT meetings—not only for people who lived in SPRs; however, it was people in SPRs who made the most of such visits. It was important that the physician should be known to the participants before the RT meeting and could communicate understandably. Interviewees in this study could see that there were opportunities to develop digital meetings, but it was clear that the need to go to the hospital in more difficult cases was obvious. Developing remote care for mild ailments was welcome. If the physician wanted to do an examination at a distance, they could contact the HCC and order an examination, and after that, the person could see their physician at a distance again. They also saw opportunities for contact via RT with the large hospital to a great extent. A participant thought that it would have been even easy to log in via an app or a smartphone:

Everything ends up on the phone, it seems, and it would have been the easiest thing to do through Messenger or whatever way you have for video, it would have been the ultimate, then you do not even have to go anywhere.

ID8; female; aged 53 years

A theme was interpreted from the 6 categories—*participation and relationships are important for good and close care via RT*—and was about people experiencing participation and relationships in different ways. When people felt involved and experienced good relationships and reliable technology, a sense of safety and security was created during the RT meeting. When patients felt less involved and the relationship or technology was not satisfactory, a feeling of distance and insecurity provided less good care via RT.

## Discussion

### Overview

In this study, we investigated the experiences of patients with cardiovascular diseases regarding follow-up meetings with their physician through RT. The inductive analysis showed a common thread throughout the categories that was interpreted as the theme, *participation and relationships are important for good and close care via RT*. Close care can mean more than just geographical proximity. The importance of relationships can be interpreted as the availability to meet those in care that we already know. A digital tool such as a video screen can also convey a sense of proximity.

Owing to the high demand for PCC, as described in the *Introduction* section, we further viewed our results considering the 6 categories developed by Sharma et al [37] in an overview of reviews of PCC. However, these components are compiled from various sources and are not mutually exclusive. Therefore, our results may fit in several of the PCC categories. We found that such an approach works well, but certain aspects of PCC in RT are missing. Therefore, we suggest that when RT is introduced, the PCC categories need to be extended with digital aspects in each of the components: *establishing a therapeutic relationship, getting to know the person*, *shared power and responsibility*, *empowering the person*, *trust and respect,* and *communication* [37]. To simplify the structure of the discussion, we abbreviate this digital extension as electronic PCC (ePCC) in analogue with eHealth.

### Principal Findings

#### Establishing a Therapeutic Relationship

##### PCC Partnership

This component is based on a partnership with mutual dependency and responsibility between the person and the professionals, and key factors are open communication, a cohesive team, and professionals who possess knowledge and skills to practice PCC [37].

##### ePCC Partnership

Our results showed that a therapeutic relationship should be established in person before an RT meeting. This relationship created security, which was confirmed by informants in the category, *safety with good relationships*, and the subcategory, *familiarity with the staff*. Some were also skeptical about RT with an unknown person, and a participant claimed, “But had it been a complete stranger then you get a little...then you keep a little distance...” Participants preferred to see a physician they were familiar with and trusted in a video consultation, which was confirmed in another study [20].

#### Getting to Know the Person

##### PCC Holistic View

This component emphasizes a holistic view of the person or patient that is more than the illness or disease that the person is diagnosed with [25,26]. It is vital for professionals to seek, understand, and acknowledge the experiences, values, and wishes of the patients and what is relevant to them. Another essential key factor is to get to know the person’s family and their culture to be able to provide care that is adapted to the patient’s need [37].

##### ePCC Holistic View

In this study, participants felt closeness via the screen and described an experience of personal contact in the category, *proximity and distance with technology*. Lavoie et al [38] believe that we enter an ethical relationship as soon as we meet a foreign face. The face expresses a meaning, and we must respond to the message of the face. For example, the experience with facial expressions described previously in this category shows that RT may work well for some. However, some other participants missed the body language and felt unsure about how to interpret the therapist’s reactions. The size of the screen and the quality of the sound and image were of great importance for how participants perceived the RT meeting. Some of the participants in this study experienced a personal contact via the screen, and some felt a distance and experienced it as impersonal, which gave them a feeling of insecurity. A feeling of alienation has been described in previous studies [17]. In the study by Shulver et al [39], some participants preferred physical visits because it was more personal, conducting the videoconference alone was isolated, and human contact was important. In our study, for example, a participant wanted a physical meeting but was never consulted. In addition, our study showed that there may be an added value for a physical meeting outside the RT meeting such as social interaction with the taxi driver or doing some shopping.

Therefore, the caregiver needs to know the person’s digital literacy and ability to communicate via ICT and whether the person has any disabilities such as visual, hearing, or cognitive impairments or those that require or which a physical meeting.

#### Shared Power and Responsibility

##### PCC Shared Responsibility

The component, *shared power and responsibility*, indicates that the patient needs to be an active part in their care, and the care delivered should be individualized and based on the person’s own needs, wishes, and values [37].

##### ePCC Shared Responsibility

There was a willingness among the patients in this study to be prepared for the RT meeting, which could be to write down questions in advance and could be seen in the category, *participation in care*, and the subcategory, *being prepared*. However, new ways of meeting could also be demanding for some people; thus, they forgot to ask questions [40], which also was described in the category, *quality of and familiarity with technology facilitating the meeting*, and participants described feelings of not being in control. Some informants felt comfortable because they were using this technology in everyday life, and others felt uncomfortable at the beginning of the meeting; however, later in the conversation, they stated that “...there was nothing strange about that.” The staff are also responsible for ensuring that the patient can use the technology before they organize an RT meeting, and they are also responsible for being able to handle the technical equipment themselves. Informants in this study commented that in some meetings, the physician was uncomfortable with the technology, and in another RT meeting, the person saw just half of the physician’s face.

#### Empowering the Person

##### PCC Empowerment

This component highlights the importance of the individual being active in their own care. The staff needs to provide patients with information, support, and resources that make it possible for them to be able to make their own decisions [37].

##### ePCC Empowerment

In this study some participants felt that the care via RT was equivalent to a physical meeting described in subcategory, *equitable care*, where patients were surprised that the digital meeting gave the same results as a face-to-face meeting, which is also described in other studies [15,20]. Participants in the study by Johansson et al [15] thought that patients would receive the same care regardless of whether they met the specialist physically or via a digital meeting. As the care is experienced as equivalent regardless of the type of visit, web-based or physical, the care becomes more accessible to the people who live in SPRs. This saves both time and money, as they do not need to travel to the HCC or hospital, and this was experienced by the individuals in the category as *good accessibility*. If the person can be in their own context where they feel safe, it is a way of empowering the individual, and the RT meeting can be as effective as a physical meeting [37].

The informants wanted to continue to meet via RT and saw opportunities to develop digital meetings further, where even mild ailments could be treated, something that could be seen in the category, *participation in care*. Another study showed that some participants had a wish for video meetings in the future, to avoid unnecessary trips to the hospital, especially because the informants became old and for other reasons [15]. Allowing patients to become involved and obtain information about how the technique works before the meeting and how this influences the development of care strengthens people’s participation in their own care [37].

#### Trust and Respect

##### PCC Personal Needs

The trust and respect component involves recognition of the person as a unique individual with their own values, preferences, lived experiences, and needs. Health care practitioners need to consider the person’s individualized needs and incorporate them into their care [37].

##### ePCC Personal Needs

In this study, the RT meetings can be equated with a physical meeting with a physician or other health care professional, where confidentiality and personal integrity are important. The informants in this study pointed out the importance of not having other people in the same room or people passing by the room in the category, *cherishing personal integrity*. For the patients who participated in an RT meeting, it was important to trust their physician, and to have a mutual respect to emphasize privacy. Patients in this study highlighted the importance of the room for digital meetings being in such a way that no other people were present except for those who were to attend the meeting, which was also found to be important in another systematic review [16]. New demands are placed on health care staff in the ambition to provide PCC for patients in connection with digital meetings, and technology provides opportunities for patients to influence future care, but there is an increased risk of integrity being violated. On the basis of our results and those of previous studies, it is important to protect personal integrity; there is always the risk of lack of confidentiality owing to a lack of control of the physical rooms where the meetings are conducted, for the patients, physicians, and technical devices used. It is even more important to decide whether it would be an RT meeting because some patients prefer to travel to a physical meeting because of social and practical needs and an added value from the journey.

#### Communication

##### PCC Information

This component about communication between the person, their family, and health professionals is important to discuss and deliver understandable and correct information about the person’s care [37].

##### ePCC Information

Communication is the most salient difference from face-to-face meeting because it is mediated through digital devices in RT. Our results showed that RT affects the person before, during, and after the meeting. This means that the other PCC components need to be taken into consideration when planning for an RT meeting.

Before the RT meeting, it is important to establish a therapeutic relationship, get to know the person, and empower the person as mentioned previously. This concerns the person’s vision, hearing, cognition, and IT literacy. Some participants in this study had hearing problems and difficulty in perceiving what was being communicated, which was experienced as insecurity. People with hearing loss may misunderstand advice or instructions, which could be prevented by using a headset or if the physician, a nurse, or an accompanying person could be present at the meeting and explain what is said [15]. The fact that people with disabilities have access to an interpreter is also a way to improve communication and promote the mutual relationship with the patient, and it is consistent with Swedish law [6]. In this study, a relative supported a person with hearing and memory difficulties—in the category, safety with good relationships.

Communication and information about how the technique works and how the meeting will be conducted are at least as important as communicating about the patient’s illness and health care for an optimal RT meeting. Some participants felt it unusual to communicate via a video screen for the first time, and some were used to it as shown in *quality of and familiarity with technology facilitating the meeting*, which was also confirmed by Johansson et al [40].

Moreover, in this study, the staff connected the device for some patients and started the meeting, which normalized the meeting and made the patients more comfortable; this is also noted in the study by Currie et al [41]. Patients felt supported when the staff connected the equipment and explained how the digital meeting would be conducted [17,20].

To master care via RT, health care professionals need to acquire knowledge and understanding about how digital technology affects the interaction between people. Technology has an important supporting function in the meeting between health care professionals and patients, but there is also a risk of the technology contributing to frustration and alienation [42].

Finally, it may be noted that an RT meeting may not be suitable for some people, as explained in the category, *proximity and distance with technology*.

During the RT meeting, it is important to share power and responsibility by ensuring technical quality and control conditions both at the caregiver’s site and at the person’s site, which may affect trust and respect.

For a person to be perceived as more sympathetic and present in a digital meeting, they should look at the camera and not at the face on the screen. The camera should also be placed at the minimum eye level; however, the distance may be less decisive [43].

In this study, it was easy for some informants to communicate in the RT meeting when the sound was good, whereas others experienced a feeling of *distance* and could not be spontaneous in the conversation; for the latter, it was difficult because they could not see body language and facial expressions, which made them feel *distant* in *proximity and distance with technology*.

Technology could also be disruptive as shown in the subcategory, *support or disturbed by technology*. An informant, for example, saw only half of the face and thought it was embarrassing to tell the physician. Similar incidents were reported by participants from another study when they could not see the computer screen because it was placed incorrectly and did not understand that they could ask for the screen to be placed differently [15]. If the picture was small and placed in a corner, the meeting felt unnatural. The use of technical equipment can be frightening for inexperienced users, and it is important to consider each person’s needs—what information and instructions they need to be able to use the new technology, so that they feel comfortable and safe.

To limit the experience of distance and isolation, the health care service can appoint a person to support the patient during the visit [38]. If the digital meeting is conducted from home, perhaps a relative can support the person or a staff member can support the person if it is from an HCC unit. Of course, no other unauthorized people should be present, as was unfortunately reported by some patients—discussed previously in the component, *trust and respect*.

Other authors believe that mobile ICT should be used with caution and that health care professionals need to understand older users, and the lack of knowledge about modern technology can affect the person’s attitude toward the new technical solutions [41,44].

After the RT meeting, it is important for the patient to feel safe, with no need to worry about the equipment. In this study, a patient “felt awfully bad...” about the fate of the equipment if it was still turned on—in the subcategory, want more information. As described in the category, participate in care, it is necessary to know the end of your responsibility when the meeting is over.

### Strengths and Limitations

Only 8 people participated in this study, which can be a weakness, but there was a diversity of sex and age represented. There were also difficulties in including more patients, as there were few patients who have had an RT meeting. However, in the remote regions and SPRs, it is a proportionally good representation because there are few people living in this area. In the last interviews, no new information emerged, which could be a sign of saturation [31]. In this study, none of the participants were younger than 53 years. On a group level, younger people may have higher digital literacy than older people. However, in PCC, group-level properties cannot be generalized to the individual. It is important to assess each person’s abilities using ICT, regardless of age, which is highlighted in our results.

Each person’s experience is unique, and there is no perfect truth to be found, but common patterns and individual differences have been reproduced and described. The results are comparable with those of previous studies with other patient experiences in digital meetings, which also confirms the transferability of the results from this study. Qualitative analysis was used because the purpose was to describe experiences of a phenomenon, a new method for meeting, and a qualitative method is therefore suitable [32]. The analysis process has been conducted close to the text with a low level of abstraction and often returned to the original material to maintain the holistic perspective, and no text materials have been excluded from the analysis [32,45]. The codes, subcategories, and categories were discussed among the authors throughout the process. A detailed description of the process and the analysis, with quotes from the interviewees, have been explained in the *Methods* and *Results* sections for a comprehensive understanding of the patients’ experiences of RT meetings and to increase the trustworthiness of the results. Our interpretation is that the categories cover the data well and can be confirmed by the quotes, thus increasing the credibility of the study.

### Implications for Future Studies and Practice

Future studies are needed to identify additional factors that affect telemedicine acceptance, such as human-technology interaction, the organization of the health care system, and social and cultural human factors. Information and education about how digital services work in practice are especially needed for patients and professionals who lack technical skills.

Patients felt that close relatives were a support, and it is important to interview relatives’ experience of RT and the staff’s perceptions. More systematic studies are needed about how people experience a digital meeting and for whom this way of meeting is suitable depending on individual conditions, resources, what disease or diseases and symptoms the person is affected by, and what the cultural context means for the willingness to seek care. For some individuals, the best way to connect to the physician may be from home. In the future, RT, in addition to patients not having to travel long distances and saving time, may also result in an economic benefit for people, communities, and health care systems as care becomes close and more accessible. Thus, there is also a great need for health economic evaluations of digital meetings in health care. It is an interesting area of research regarding what savings can be made in terms of climate impact and sustainable development when travel is reduced.

Finally, as technical development is exponentially fast and both professionals and older people will probably have better skills, high digital literacy, and more experiences with various platforms, the results of this study may not be repeatable over time; however, other factors may turn out to be as important for optimal RT meetings.

### Conclusions

This study has shown that *participation and relationships are important for good and close care via RT*. To improve the quality of an RT meeting, PCC can be applied but needs to be extended to ePCC, especially the communication component as the most salient difference from a face-to-face meeting.

*Before* an RT meeting it is crucial to do the following:

1. Establish a *therapeutic relationship*, *get to know the person*, and *empower the person*, preferably at a previous physical meeting.
2. Decide whether the meeting should be held in person or via RT based on the person’s preferences, abilities, and social and practical needs.
3. If conducting an RT meeting, get to know the person’s vision, hearing, and cognitive abilities and digital literacy and take measures, if necessary.
4. Acquire knowledge and understanding of how digital technology works and how to manage it among the health care staff.

*During* the RT meeting it is vital to do the following:

1. Ensure technical quality and control conditions both at the caregiver’s site and at the person’s site.
2. Ensure that the meeting rooms are designed in a safe and secure manner and that privacy and confidentiality are ensured.
3. Place the camera at the minimum eye level.
4. Look at the camera and not at the face on the screen.

*After* the RT meeting, it is important to do the following:

1. Ensure that the patient feels safe and that they do not need to worry about the equipment.

RT meetings need to be created with various actors within the care organization based on a person-centered approach, where the patient is a cocreator of good and close care in the future.

## Abbreviations

COREQ: Consolidating Criteria for Reporting Qualitative Research
DHR: digital health room
ePCC: electronic person-centered care
HCC: health care center
ICT: information and communication technology
PCC: person-centered care
RT: remote treatment
SPR: sparsely populated region
